# A Field Experiment on Reducing Drinking Straw Consumption by Default

**DOI:** 10.3389/fpsyg.2020.565537

**Published:** 2020-09-16

**Authors:** Daria Mundt, Sebastian Carl, Nico Harhoff

**Affiliations:** Institute of Psychology, University of Kassel, Kassel, Germany

**Keywords:** green nudges, default choice, decision making, plastic waste, environmental behavior

## Abstract

Against the background of the pollution of the environment through plastic waste, we conducted a field experiment (*N* = 195) to test the effectiveness of a default nudge intervention that aimed at reducing the consumption of plastic drinking straws. We assumed that separating straws from cups by default leads to an overall decrease in straw consumption. We hypothesized that individuals would consume straws less frequently when they had to pick straws actively out of a separate straw box for their drink compared to when they could choose between cups already containing and not containing straws. Results of a logistic regression revealed a significant difference between both conditions concerning the use of drinking straws [*B* = 1.129 (*SE* = 0.30), *p* < 001 with an odds ratio of *OR* = 0.32]. Confirming our hypothesis, results underline that minor and subtle interventions addressing waste reduction might have marked effects. More research is needed to improve current and future interventions to significantly reduce the amount of plastic consumption and consequently reduce the waste in the environment.

## Introduction

Plastics are estimated to make up approximately 10% of global waste (Barnes et al., 2009) and more than 80% of marine litter (European Commission, 2018). About 70% of all marine litter items are – beside lost and abandoned fishing gear – single-use plastic products, such as straws, tobacco product filters, food containers, balloons, and sticks for balloons (European Commission, 2018). The current use and disposal of plastic affect the environment, including the pollution of soil (Bläsing and Amelung, 2018), water, and sea (Li et al., 2016). Plastic pushes climate change (Royer et al., 2018; Center for International Environmental Law, 2019) and endangers the human health (Rist et al., 2018). To reduce littering and avoid environmental damage, the European Parliament approved a law for 2021, banning 10 single-use plastic items such as plates, cutlery, straws, and cotton bud sticks (European Parliament, 2019). The ban’s impact is proposed to reduce littering by more than half and is expected to avoid the emission of 3.4 million tons of CO_2−e_ by 2030 (European Commission, 2018).

Bans are, by their nature, effective political measures to prevent unwanted practices (Heidbreder et al., 2019). However, bans may have unintended consequences. For example, citizens could respond with reactance. If individuals perceive a threat or even an elimination of their behavioral freedom, they try to restore this particular threatened freedom. For example, the lost choice alternative becomes more attractive, whereas the forced choice is becoming less attractive (cf. the reactance theory by Brehm, 1966). To counter reactance, the use of *nudges* can be considered. A nudge intervenes in people’s choice architecture, adjusting people’s behavior in a predictable way. Thus, nudges are small changes in the environment, triggering heuristic processes in individuals, which, in turn, lead to a particular choice of behavioral option. To count as a nudge, this intervention must not forbid other choice alternatives or change the economic cost of a behavior significantly (Thaler and Sunstein, 2008).

Nudges take advantage of cognitive, social, and moral factors that underline human decision making (Moseley and Stoker, 2013). For instance, some nudging strategies make use of social norm heuristics, for example, by comparing consumer energy usage with the average usage of their neighborhood (Allcott and Rogers, 2014). Other strategies increase the salience of the aimed choice alternative. For example, Kurz (2018) reduced meat consumption in a university restaurant by placing the vegetarian meal at the top position of the restaurant’s menu and by presenting the dish to consumers at a visible spot.

Another nudging strategy is changing the default when people have to make a decision (Vetter and Kutzner, 2016). In decision situations, people tend to choose the alternative that demands less effort from them. Therefore, desirable behavioral options can be implemented as a default option, which is the behavioral option, people automatically receive if they do not change anything (Dinner et al., 2011). For example, Chapman and Ogden (2012) found that when offering brown bread for sandwiches only, consumers bought more brown than white bread. Egebark and Ekström (2016) reduced a university’s paper consumption by changing the default setting of the printer to double-sided printing.

Changes in default can also have positive effects on reducing the consumption of drinking straws. Wagner and Toews (2018) analyzed self-reported data of 133 gastronomies that introduced as a default option only drinks without straws. To receive a straw, clients had to ask explicitly for it or take the straws out of a box by themselves. This intervention led to a reduction in straw consumption by 32% on average compared to the time before this default option was introduced. Some clients criticized this intervention negatively, emphasizing the danger of reactance in consumers.

To avoid negative reactions on a default option aimed at reducing straw consumption, we developed an experimental pattern in which the default option was more discreet. We offered drinks and straws separately but with the same distance to the consumers. Our field experiment aimed at providing further empirical evidence for using default options as a nudge to reduce straw consumption as one of the most used and polluting single-used plastic products. According to the findings of Wagner and Toews (2018) and research on the default effect, we expected individuals to choose drinks without straws when they have to pick the straws actively. More specifically, we hypothesized that individuals would consume straws less frequently when they had to pick straws for their drink actively out of a straw box separate from the cups compared to when they could choose cups already containing the straws.

## Materials and Methods

### Participants

The sample consisted of *N* = 195 participants with 96 participants in the experimental condition and 99 participants in the control condition. Participants did not know that they were part of an experiment. Therefore, the demographic data of participants were predicted by the experimenters. A total of 95 participants were estimated as 14–30 years old, 75 persons were estimated as 31–60 years old, and 25 persons were estimated as above 60 years of age. Of the rated participants, 103 were female and 92 were male. We excluded participants from our sample who were not able to choose their lemonade on their own (e.g., due to a disability or interference from parents in the case of children). Furthermore, we excluded participants who had to use straws because of health reasons and participants who tasted more than one offered cups of lemonade.

### Design and Procedure

The study was based on a one-factorial design with the independent variable *Nudge* with two manifestations. In both conditions, participants were invited to choose out of several cups filled with approximately 75 ml of self-mixed lemonade. In the experimental condition, cups were presented without straws. However, straws could be taken actively out of a coverless box offered beside the cups. In the control condition, the straws were already inside half of the offered cups. The other half of the offered cups did not contain straws. The dependent variable was the choice for or against straw consumption.

The study was conducted in three different locations in the city of Kassel, Germany. Due to the differing location settings, we had to change slightly some characteristics of the experiment. The first assessment was conducted in the evangelical family education center “Katharina-von-Bora Haus.” In the foyer of the building, we set up a table at which we offered the lemonade to the center’s visitors. Since we had access to the kitchen, we used glass cups for the lemonade. In order to cover up the experimental situation, we pretended to do an advertising campaign for the upcoming festival of the city’s university by displaying several flyers and providing further verbal information if asked for. Due to recurring periods with no potential participants in the foyer, we shifted the data collection to the surrounding of the education center. Therefore, we approached people passing by the street with the filled glass cups on a serving tray with a comparably smaller number of glasses as inside the education center. Furthermore, we adapted the cover-up story by asking participants to rate the taste of the lemonade. The third assessment was carried out at the summer festival of the University of Kassel. The setup and cover story were the same as in our second location, outside the education center. As we did not have access to a kitchen, we had to use plastic cups instead of glasses to serve the lemonade.

In order to ensure the random assignment of the participants to the two conditions, we switched the experimental setup between the two conditions on a regular basis. Depending on the current number of potential participants, we defined an approximate number of people (between 20 and 50) before each trial, to mark the time after which the condition had to be changed. Upon reaching half of that number, the positions of the cups were changed within one condition. In the control condition, we rearranged the cups with straws, for example, from the right to the left side of the table or tray. Respectively, we moved the straw box from the one to the other side of the offered cups in the experimental condition.

## Results

Analyses were performed with RStudio (version 1.1.463), using the R packages car (Fox and Weisberg, 2019), mlogit (Croissant, 2019), dplyr (Wickham et al., 2019), and ggplot2 (Wickham, 2009). The analyses employed an alpha level of 0.05 (two tailed).

Since both the independent and dependent variables were binary, we conducted a binomial logistic regression. The independent variable Nudge was coded 0 = control condition and 1 = experimental condition. The dependent variable was coded 0 = straw consumption and 1 = straw avoidance.

**Figure 1:**
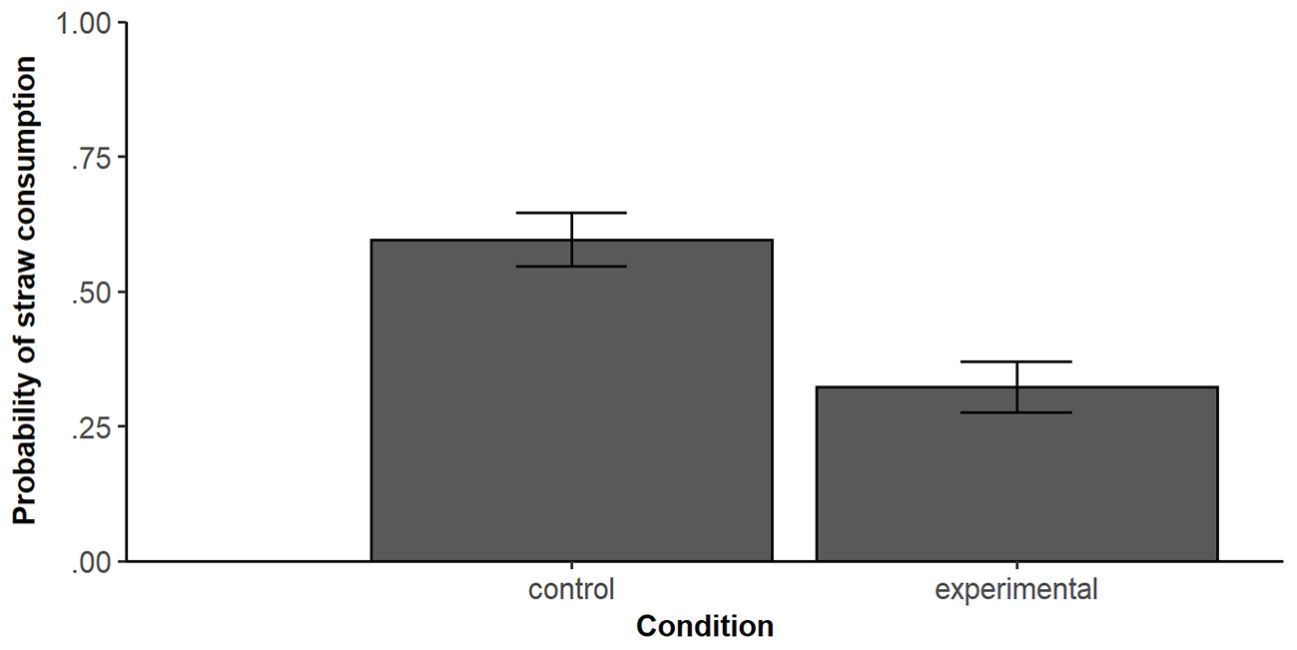
Estimated marginal means (EMMs) for the probability of consuming a drinking straw for the control and experimental conditions. *N* = 195. Error bars represent ± standard error of the EMMs.

The binomial logistic regression showed a significant difference between both conditions concerning the use of drinking straws [*B* = 1.129 (*SE* = 0.30), *z* = −3.77, *p* < 0.001 with an odds ratio of *OR* = 0.32, 95% *CI* (0.178, 0.577)]. The converted *OR* results in Cohen’s *d* = −0.63, which can be conceived as a medium effect. Model comparison with an intercept-only model revealed a significant fit of the model [*χ* ^2^ (1) = 14.82, *p* < 0.001]. Figure 1 visualizes higher probabilities to consume straws in the control condition. The depicted estimated marginal means (EMMs) of the probabilities for both conditions were calculated using the R packages emmeans (Lenth, 2019).

A *post hoc* power analyses with G*Power (version 3.1.9.4; Faul et al., 2007) revealed a high power of 0.96 for the logistic regression. Type-1 error probability was set to 0.05, and effect size (*OR*) was set to 0.32. Furthermore, we assumed a baseline of 0.50, no covariates, binomial distribution, and a target of an even distribution of participants in both conditions.

To check for possible effects of our differing experimental settings on the straw consumption, we conducted several exploratory analyses. The full results are reported in Table 1. Logistic regressions with the single-predictor type of cups, offering type (table or serving tray), and location, revealed only a significant effect for the location, in particular, for the university festival compared to the foyer of the family education center [*B* = −0.76 (*SE* = 0.37), *z* = −2.04, *p* = 0.041 with *OR* = 0.47, 95% *CI* (0.23, 0.97)]. Thus, visitors of the university festival were less likely to choose a straw, compared to the visitors of the family education center. However, a model comparison with the intercept-only model revealed a nonsignificant fit of this model compared to an intercept-only model.

**Table 1:** Estimated coefficients (*B*), standard errors (*SE*), information criteria for regression models, and comparisons.

Fixed effects	Model 0		Model_Location		Model_Cup		Model_Serving	
	*B*	*SE*	*B*	*SE*	*B*	*SE*	*B*	*SE*
(Intercept)	−0.15	0.14	0.30	0.30	0.07	0.19	0.30	0.30
Location 2: surrounding compared to foyer			−0.36	0.39				
Location 3: summer festival compared to foyer			−0.76	0.37^*^				
Cup: plastic compared to glass					−0.53	0.30		
Serving: tray compared to table							−0.60	0.34
Information criterion								
AIC	271.17		270.85		269.90		270	
Residual deviance	269.17		264.85		265.90		266.00	
Residual df	194		192		193		193	

* *p* = 0.041.

*N* = 195; *n_experimental_* = 96; *n_control_* = 99. Model 0 = intercept-only model with the choice of straw consumption as binary dependent variable. Model_Location = Model 0 + location as dummy-coded independent variable with dummy Location 2: surrounding of education center compared to foyer (reference group) and Location 3: summer festival compared to foyer (reference group); Model_Cup = Model 0 + Type of cups as independent variable with 0 = glass cup and 1 = plastic cup. Model_Serving = Model 0 + type of serving as independent variable with 0 = table and 1 = serving tray. Model comparison of Model 0 with: Model_Location *χ* ^2^ (1) = 4.32, *p* = 0.12.; Model_Cup *χ* ^2^ (1) = 3.27, *p* = 0.070; Model_Serving *χ* ^2^ (1) = 3.17, *p* = 0.075.

## Discussion

In the present field experiment, we examined the effect of an easy-to-implement default-nudge intervention on reducing drinking straw consumption. Due to the widespread standard in gastronomy that drinks already contain a straw when they are served, we assumed that a separation of both components (i.e., cups and straws) by default leads to an overall decreased straw consumption. Our hypothesis was confirmed: individuals consumed less straws when they were offered in a separate box next to cups without straws as a default option, compared to when participants could choose out of cups already containing straws. Our results corroborate the findings of Wagner and Toews (2018), who found a reduction in straw consumption after the implementation of a default nudge in restaurants. Thus, drinks were not served with straws anymore. To receive a straw, clients had to ask explicitly for it or take the straws out of a box placed in a specific place in the restaurants by themselves. However, some clients felt offended by this intervention, emphasizing the danger of reactance toward this nudging strategy in consumers. To avoid negative reactions on a default option aimed at reducing straw consumption, we developed an experimental pattern in which the default option was more discreet. Drinks were also served without straws, but the straw box was placed near the cups. By doing so, consumers had access to both behavioral options (straws or no straws), but were still nudged toward consuming the lemonade without a drinking straw.

The limitations of this study are worthy of consideration. To start with, there might be a bias in the straw consumption induced by other participants and the experiment itself. As we offered drinks in public, we could not control any effects of the social norms introduced by the group members that served themselves at the same time. Thus, individual decision-making processes are also influenced by social heuristics (Venkatesan, 1966; Cheung et al., 2017). It would be interesting to analyze the possible effects of peers in decision making regarding straw consumption to gain better insight into the effectivity of our default nudge. Moreover, peer pressure induced by the experimenters, themselves, might have influenced the participants. In the control condition, the participants were clearly exposed to the two options *straw* or *no straw*, which might have activated the social norm against plastic straws to a high degree. In the experimental condition, the social norm against straws might have been activated, as taking a straw out of the box demands effort to resist the social norm (cf. Bamberg and Möser, 2007). Investigating the effect of social norms in default nudge interventions should be considered to give a clearer insight in further mechanisms underlying the nudge effect.

Another limitation concerns our experimental setting, which we had to adapt several times due to external circumstances. We used different cups, offered the drinks in different settings (table or serving tray), and changed the cover story depending on the location. However, exploratory analyses revealed no influence of these variables on straw consumption. Nevertheless, replications of the present study in constant settings are important to generalize our finding. Especially in more typical drink-serving contexts (e.g., restaurants, bars), a replication needs to be considered. Thus, in our case, people received the drinks for free. Moreover, our lemonades were not drinks for which drinking straws had any important or necessary functional role in consumption. Thus, consumers without specific disabilities could easily renounce the use of straws without losing the capability of consuming their drink. In contrast, straws in cocktails or long drinks are also used to stir and separate liquids from other ingredients. A focus on drinks that typically contain straws, such as cocktails, and its comparison to drinks that are not expected to contain straws is important to get clearer insights on the effect of default nudges in the context of straw consumption.

There are many alternatives to one-way plastic drinking straws, such as glass, stainless steel, bamboo, or paper straws (e.g., Zanghelini et al., 2020). However, these alternatives are not necessarily more environmentally friendly than plastic straws. Thus, the whole life cycle of a product needs to be considered. Therefore, the best would be to avoid using straws (if not necessary for consumers with disability or medical reasons; Zanghelini et al., 2020). Default nudges can help to wean people from a product that is not essential for consumption.

In this context, the effect of prompts should be considered. Fritz et al. (2017) showed that signs prompting recycling can increase recycling behavior even when decreasing the proximity of the bins. Therefore, offering glasses without straws as a default option with the separately offered straws in a box, and provided with prompts that inform about the impact of straw consumption, might be an effective strategy to significantly lower straw consumption and lower the discontent about missing straws as reported by Wagner and Toews (2018).

Generally, when aiming at sustainable changes in people’s behavior, a combination of nudge interventions and informal campaigns seems to be important. Synthia and Kabir (2015) found that consumers used increasingly new alternative bags after the ban of plastic bags and wrongly assumed that the alternatives of plastic bags are, by nature, more environmentally friendly. Therefore, it is important to provide information to consumers when nudging them away from one behavior to another.

Raising consumers’ awareness about the negative impact of plastic consumptions is also important to increase the effectiveness of nudge interventions, which depends also on consumers’ acceptance of nudge implementations. People might react with reactance to nudging when perceiving the intervention as an attempt to control them or as paternalism (Sunstein, 2017). However, if they believe that the nudge fulfills a legitimate purpose that fits their interests and values, they accept this intervention (Reisch and Sunstein, 2016; Schubert, 2017).

## Conclusion

The present study emphasized the potential of implementing default choice as an effective method to lower straw consumption. Potentially, this effect can be extended to other environmentally damaging consumptions, too (e.g., single-use plastic cups or cutlery). Using nudges as a political measure to achieve the targets of consumption reduction as suggested by the European Commission (2018) can be a promising approach. Bans or the introduction of fees can be accompanied by nudge interventions to make people incorporate pro-environmental behaviors. When consumers are already used to consume less or no plastic straws, the risk for reactance as a result of the prohibition planned by governments can be reduced. More research is needed to improve current and future interventions to significantly reduce the amount of plastic consumption and, consequently, waste in the environment.

## Data Availability Statement

The dataset and R script presented in this study can be found in online repositories: https://osf.io/nxdje/.

## Ethics Statement

Ethical review and approval was not required for the study on human participants in accordance with the local legislation and institutional requirements. Written informed consent from the participants’ legal guardian/next of kin was not required to participate in this study in accordance with the national legislation and the institutional requirements.

## Author Contributions

DM, SC, and NH: conceptualization, methodology, formal analysis, method, and results. DM: original draft preparation – theory and discussion. All authors contributed to the article and approved the submitted version.

### Conflict of Interest

The authors declare that the research was conducted in the absence of any commercial or financial relationships that could be construed as a potential conflict of interest.
